# Influence of scattered trees in grazing areas on soil properties in the Piedmont region of the Colombian Amazon

**DOI:** 10.1371/journal.pone.0261612

**Published:** 2021-12-29

**Authors:** Faver Álvarez, Fernando Casanoves, Juan Carlos Suárez

**Affiliations:** 1 Programa de Medicina Veterinaria y Zootecnia, Facultad de Ciencias Agropecuarias, Universidad de la Amazonia, Florencia, Caquetá, Colombia; 2 Laboratorio de Evaluación de Forraje y Nutrición Animal, Centro de Investigaciones Amazónicas CIMAZ Macagual, Universidad de la Amazonia, Florencia, Caquetá, Colombia; 3 Grupo de Investigación en Sistemas Agroforestales para la Amazonia -GISAPA, Universidad de la Amazonia, Florencia, Caquetá, Colombia; 4 CATIE-Centro Agronómico Tropical de Investigación y Enseñanza, Turrialba, Costa Rica; 5 Maestria Sistemas Sostenibles de Producción, Facultad de Ciencias Agropecuarias, Universidad de la Amazonia, Florencia, Caquetá, Colombia; 6 Centro de Investigaciones Amazónicas CIMAZ Macagual, Grupo de Investigaciones Agroecosistemas y Conservación en Bosques Amazónicos-GAIA, Florencia, Caquetá, Colombia; 7 Programa de Ingeniería Agroecológica, Facultad de Ingeniería, Universidad de la Amazonia, Florencia, Caquetá, Colombia; Fujian Normal University, CHINA

## Abstract

Trees dispersed in grazing areas are contribute to the sustainability of livestock systems. The interactions between trees and soil are ecological processes that allow the modification of the biology, fertility, and physics of the soil. This study was aimed to assess the influence of dispersed trees in pastures on soil properties in grazing areas for dual-purpose cattle systems in the Piedmont region of the Colombian Amazon. The work was done in grazing areas with scattered trees at the Centro de Investigaciones Amazónicas CIMAZ–Macagual in Florencia—Caquetá—Colombia. We evaluated the effect of five tree species, *Andira inermis*, *Bellucia pentámera*, *Guarea Guidonia*, *Psidium guajava* and *Zygia longifolia*, on soil properties (up to 30 cm soil depth) under and outside the influence of the crown. Under the tree crown, three points were systematically taken in different cardinal positions. This was done at a distance corresponding to half the radius of the tree crown. The sampling points in the open pasture area (out of crown) were made in the same way, but at 15 m from the crown border. The ANOVA showed significant interaction (P < 0.0001) between tree species and location for macrofauna abundance up to 30 cm soil depth. For this reason, we performed the comparison between locations for each tree species. Chemical soil variables up to 10 cm soil depth only showed interaction of tree species-location for exchangeable potassium (P = 0.0004). Soil physical soil characteristics up to 30 cm soil depth only showed interaction of tree species-location at 20 cm soil depth (P = 0.0003). The principal component analysis for soil properties explained 61.1% of the total variability of the data with the two first axes. Using Monte Carlo test, we found crown effect for all species. Trees help to control exchangeable mineral elements that can affect the soil, potentiate basic cations such as magnesium and potassium, increase the abundance of soil macrofauna; but some trees with high ground level of shade in grazing areas could increase soil compaction due to the greater concentration of cattle in these areas.

## Introduction

The department of Caquetá is located in the northwest of the Amazon region. It has an area of 89,530 km^2^ that corresponds to 7.8% of the country’s continental surface; it is part of the so-called Western Amazon subregion of Colombia [1]. In this region, livestock activity occurs in all types of farm landscapes, whose sizes vary from 30 ha to more than 500 ha, with an average of 120 bovines per farm [2]. Historically, there were changes in land use cover that started from the forest to agricultural production systems (annual crops, palm, and pastures) which were sponsored by state policies. This process has been called by some authors as the empowerment of the Amazon, causing the loss of ecosystem services, as well as reduced livestock systems efficiency [3].

In this context, deforestation and the conversion of Amazonian forests into pastures and croplands can have negative effects on the soil due to an excessive stocking, turning grazing areas into degraded pastures [4]. This practice has increased compaction, erosion, nutrient depletion, and general loss of soil fertility and biodiversity [5–8]. The soil macrofauna in particular has proven to be a sensitive indicator of the alteration of the vegetation cover [8–12], and it can considerably affect the decomposition and cycling processes of soil nutrients [13–15].

The conservation of the tree component in grazing areas allows transforming traditional livestock systems into more sustainable productive systems [3]. In addition, it is important to know the effect of scattered trees in pastures on soil properties [16, 17], ecosystem services [18] and animal production [19, 20]. The presence of trees in pastures have several agroecological advantages [21]. These include a higher content of soil nutrients, although well below that of the amount stored in the natural forest [22] and the growth of tree seedlings and crop species under its crown [23]. Likewise, the distribution and nature of plants in an agroforestry system greatly influence soil biology [24–26]. The particular trophic and microclimatic conditions in the vicinity of the trees can affect the abundance and richness of soil macrofauna [27].

Scattered trees in paddocks contribute to improve soil fertility [28], but the level of improvement depends on the tree species and functional traits such as leaf type and size, crown size, and architecture, among others [29], their arrangement and spatial distribution, and the management given to the scattered trees [30, 31]. Knowing the impact of scattered trees in pastures on soil characteristics (biological, chemical, and physical) will contribute to better pasture management decisions including animal stocking rate applied. This is, because pasture and tree biomass production depends on changes in soil fertility [32]. The objective of this work was to determine the effect of the most common tree species scattered in pastures on the biological, chemical, and physical properties of the soils in grazing areas of managed dual-purpose cattle systems in the Piedmont region of the Colombian Amazon. We expect to find a significant effect of tree canopy on soil variables, with the effect of some tree species on biological, chemical, and physical variables being more significant. We tested the two specific hypothesis: *i)* tree canopy has a significant effect on soil variables, and *ii)* tree species common of cattle farms in the Colombian Amazon region influence soil properties.

## Materials and methods

### Study area

The study was carried out at the Centro de Investigaciones Amazónicas CIMAZ–Macagual "Cesar Augusto Estrada González", located 22 km from Florencia, a city in the south of the Caquetá department—Colombia, with about 380 ha for livestock production. It is geographically located in the Colombian Amazon at 1° 37 ’N and 75° 36’ W, at 300 m above sea level, and Afm type (Warm-Humid Tropical Forest) in the climatic classification according to Köppen [33]. The area presents average annual precipitation of 3,793 mm, a solar brightness of 1,707 h year^-1^, an average temperature of 25.5°C and relative humidity of 84.25%. It is located within the life zone of the Tropical Humid Forest (Bh-T) defined by Holdridge [34].

### Tree selection and sampling points

Five tree species *Andira inermis*, *Bellucia pentámera*, *Guarea Guidonia*, *Psidium guajava* and *Zygia longifolia* were selected to evaluate the influence of scattered trees on the soil properties in grazing areas of the managed pastures. These species were selected because they were the ones that presented the highest value index of ecosystem importance in a census of 4,657 trees in the cattle farms in the Colombian Amazon region [35]. Except for *Guarea guidonia*, they were the most named by the cattle producers of the region in a study on local knowledge and provision of ecosystem services [35]. Two individuals of each tree species that did not have overlapping crowns and shade areas to ensure the independence of the observations were randomly selected within the paddocks as replications. Individuals within a tree species were similar in architecture, shape, height, and crown size. In each individual tree, three sampling points were taken in the North, East and West position in the middle of the radius of the crown (distance below the crown). The sampling points in the open pasture area (outside of crown) were carried out in a North, East, and West position, 15 m from the edge of the crown of each individual tree (distance out of crown) selected for soil sampling.

### Evaluation of soil biological, chemical, and physical characteristics

We evaluated biological, chemical, and physical characteristics of soils sampled under and outside tree effect in all sampling points. For the characterization of the soil macrofauna, we used the ISO 23611–5 standard [36]. A soil monolith was taken (25 × 25 cm at a soil depth of 30 cm) and for the extraction of this monolith we used a metallic angle frame. For each tree and distance to the crown of the tree (below or outside), all the fauna of the soil in the young and adult stage found in the litter, and in the 30 cm of soil was taken without differentiating by depth, for a total of 60 data (5 species × 2 repetitions × 2 distances to the crown of the tree × 3 positions). The collected fauna samples were placed in plastic bottles with 97% alcohol, and later a morphological description and a taxonomic classification were made at the order level of the individuals found.

For chemical characterization of the soil, a composed sample of three cardinal positions at 0–10 cm depth was taken for each tree and distance to the crown of the tree (below or outside) obtaining 180 observations (5 species × 2 repetitions × 2 distances to the crown of the tree × 3 positions × 3-fold soil lab determinations). The three lab determinations were averaged to yield n = 60 data points. For each soil sample we determined: *i*. soil organic carbon (SOC) by oxidation of dichromate [37] in an acid medium for 30 min in a digester block at a constant temperature of 155°C, and titration of the non-oxidized dichromate employing Mohr’s salt; *ii*. Soil organic matter (SOM) was estimated by multiplying the SOC value with 1.7 [38]; *iii*. exchangeable cations (Ca^2+^, Na^+^, Mg^2 +^, K^+^) and exchangeable aluminum (Al ^3+^) extracted by successive washes with a 0.2 N BaCl solution in an extract-soil ratio of 1:5 following the method of Mehlich [39] with the modification highlighted by Lax et al. [40]. The concentrations of the exchangeable cations and Al were determined by Ion Chromatography (IC) in an accredited laboratory; i*v*. estimation of aluminum saturation (AlS); *v*. available inorganic phosphorus (P) extracted by the Olsen method [41] using bicarbonate (0.5M NaHCO_3_) at pH 8.5, in a solution-soil ratio of 1:20 [42]. The phosphorus resulting from the extracts, previously neutralized with a dilute HCl solution, was determined calorimetrically by the ascorbic acid method according to Murphy and Riley [43]; *vi*. potentiometric hydrogen potential (pH); and *vii*. cation exchange capacity (CEC).

For characterization of soil physical characteristics, we used a 50 × 50 × 50 cm test pit sampling in the three positions (N, W, E) at 0–10, 10–20, and 20–30 cm depth for each tree and distance to the crown of the tree (below or outside), obtaining 60 observations (5 species × 2 repetitions × 2 distances to the crown of the tree × 3 positions) for each of the three depths. Using the volume cylinder method [44] (98.1 cm^3^) the percentage of moisture and the bulk density of soil were determined. Likewise, the soil resistance to penetration was determined using a hand penetrometer model 0601 (Eijkelkamp Agrisearch Equipment, Giesbeek, The Netherlands).

Data were analyzed through an analysis of variance with linear mixed models (LMM) for continuous variables and generalized linear mixed models (GLMM) with a Poisson distribution for the abundance and richness of macrofauna orders. The model considered the fixed effects of the tree species, distance to the crown of the tree (below or outside), and the interaction species by distance to the crown, and the random effects of tree [45] and position within tree. To determine differences between treatment means, Fisher’s LSD test was used (p <0.05) and in cases where there were interactions between species and distance factors, orthogonal contrasts were used to determine differences between distance within each species. The analysis was performed using the InfoStat program [46] and its interface to R [47].

Principal component analysis (PCA) was carried out to explore the relationship between biological variables, and between chemical and physical variables and to determine multivariate differences between tree species and association among variables. Significance was tested using a Monte Carlo test (1,000 simulations). PCA allows to analyze the interdependence of metric variables and to find an optimal graphical representation of the variability of the data in a table of n observations and p columns or variables. This exploratory analysis tries to find, with minimal loss of information, a new set of uncorrelated variables (principal components) that explain the structure of variation in the rows of the data table. Additionally, co-inertia analysis was used to explore covariation and general similarity in data structure between the soil biological, chemical, and physical data sets. Multivariate analysis was performed in R.3.4.4 software [47], using the *Ade4* package.

## Results

### Soil biological characteristics

**Fig 1:**
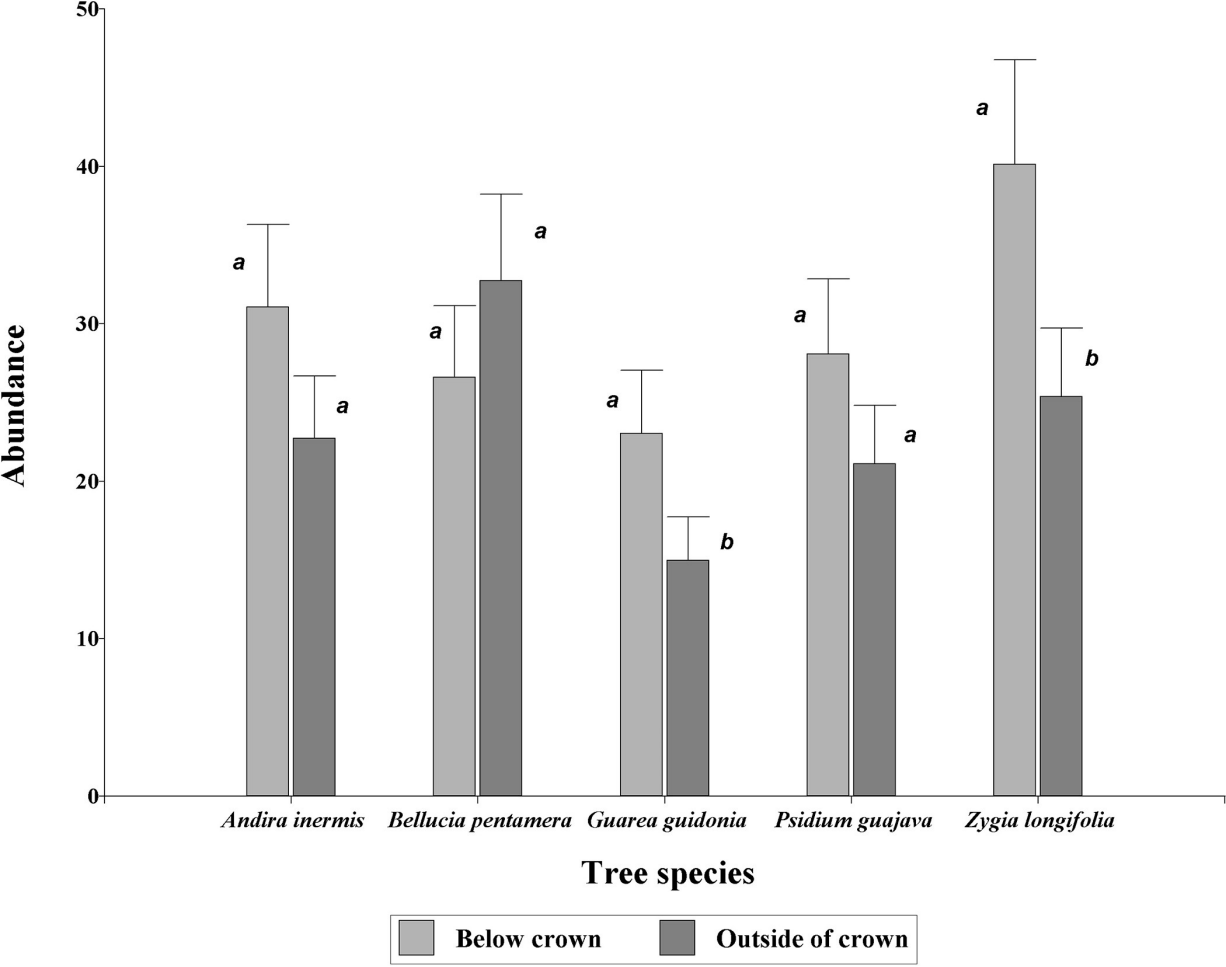
Abundance of macroinvertebrates below and outside the crown of tree species in grazing areas of pastures in the Colombian Amazonian piedmont. Equal letters within a species indicate equal means (P > 0.05).

The analysis of variance using a GLMM showed highly significant differences (P <0.0001) for the abundance of macroinvertebrate orders between distance to the crown of the tree (below or outside) (29.26 ± 2.22 and 22.66 ± 1.77 below and outside of crown, respectively, Fig 1). However, the model showed significance in the interaction of tree species by distance (P = 0.0004), for which the positions within each tree species were compared. *Guarea guidonia* and *Zygia longifolia* presented higher abundance below the crown (40.14 ± 6.62 for *Z*. *longifolia* and 23.04 ± 4.01 for *G*. *guidonia*) than outside the crown (25.38 ± 4.36 for *Z*. *longifolia* and 14.97 ± 2.76 for *G*. *guidonia*) (Fig 1). *Andira inermis*, *Psidium guajava*, and *Bellucia pentamera* did not show differences between locations.

For the variable on richness of macroinvertebrate orders, there was no interaction between tree species and distance (P = 0.5831), and there were no differences between the two distances (P = 0.6557). But there were differences between tree species (P = 0.0378), where *A*. *inermis* was the one with the highest richness (4.82 ± 0.63), followed by *Z*. *longifolia* (4.52 ± 0.62), *B*. *pentamera* (4.14 ± 0.59), *P*. *guajava* (2.91 ± 0.49), and ending with *G*. *Guidonia* (2.74 ± 0.48) (Table 1).

**Table 1:** Macroinvertebrate richness in scattered trees in grazing areas of pastures in the Colombian Amazonian piedmont.

Tree species			p-value
	Mean	S.E.	
*Andira inermis*	4.82	0.63a	0.0378
*Bellucia pentamera*	4.14	0.59ab	
*Guarea guidonia*	2.74	0.48b	
*Psidium guajava*	2.91	0.49b	
*Zygia longifolia*	4.52	0.62a	

Equal letters between species indicate equal means (P > 0.05).

### Soil chemical characteristics

**Fig 2:**
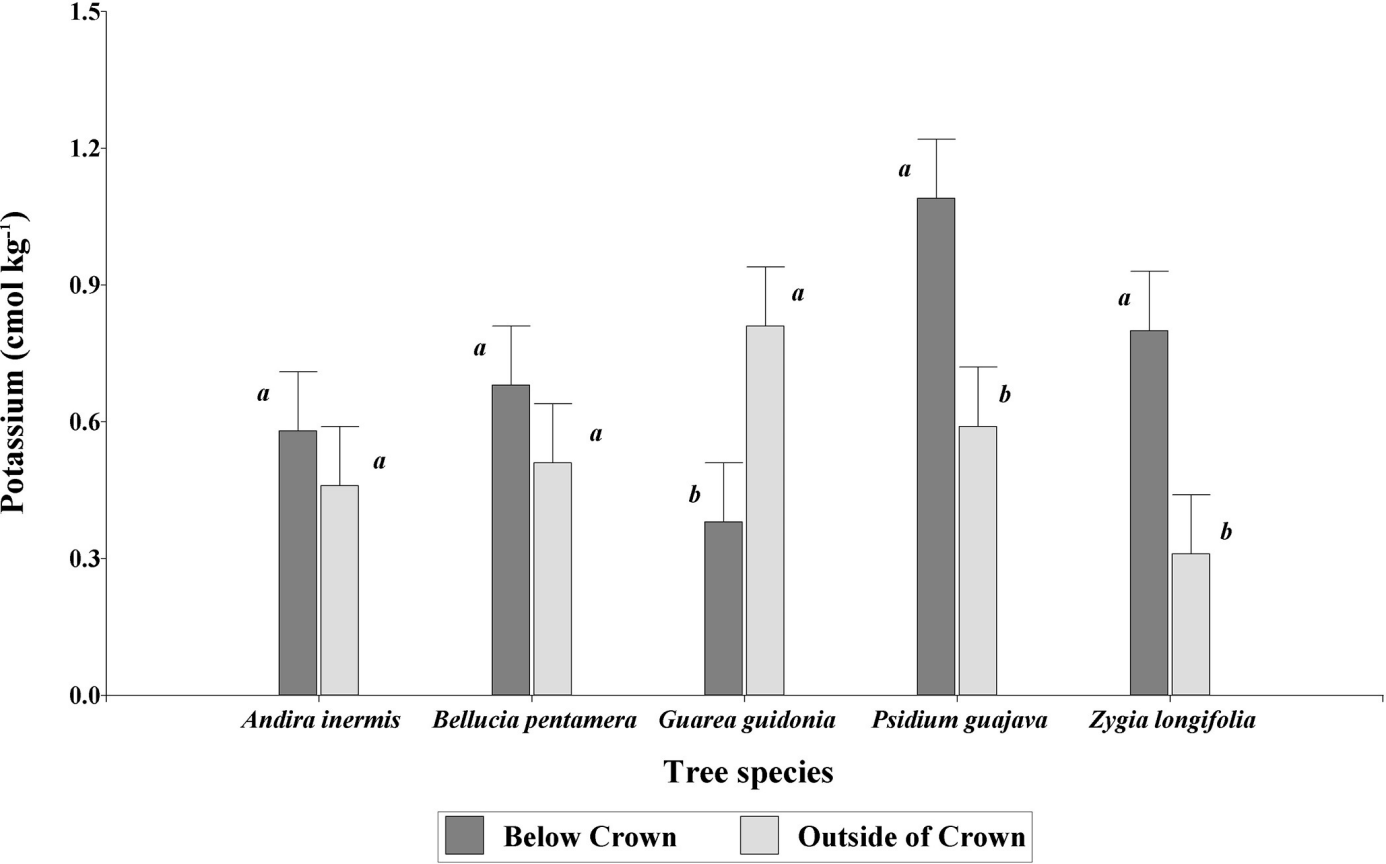
K^+^ content in species-by-distance interaction (below and outside of crown) of scattered trees in grazing areas of pastures in the Colombian Amazonian piedmont. Equal letters within species indicate equal means (P > 0.05, n = 18).

For the chemical characteristics evaluated, the analysis under the LMM only showed significant interaction (P = 0.0046) between tree species and distance for the K^+^ variable. Differences were found between below and outside of crown (p = 0.0410), with the highest average content of K^+^ under the crowns (0.71 ± 0.06 cmol kg^-1^) than outside these (0.54 ± 0.06 cmol kg^-1^). *Psidium guajava* and *Z*. *longifolia* presented higher K^+^ content below the crown (1.09 ± 0.13 cmol kg^-1^ and 0.80 ± 0.13 cmol kg^-1^ respectively) than outside of crown (0.59 ± 0.13 cmol kg^-1^ and 0.31 ± 0.13 cmol kg^-1^ respectively) (Fig 2). For *B*. *pentamera* and *A*. *inermis*, there were no differences between distance. *Guaea guidonia* presented differences between distance, but here the highest K^+^ content occurred at the outside of crown distance (0.81 ± 0.13 cmol kg^-1^) and not under the crown distance (0.31 ± 0.13 cmol kg^-1^) (Fig 2).

The rest of the soil chemical variables evaluated did not present significant differences for tree species by distance interaction and the distance effect. Thus, only results on differences between tree species are presented. For soil pH, analysis showed differences (P = 0.0337) between species with *A*. *inermis* and *Z*. *logifolia* presenting highest mean values (5.42 ± 0.14 and 5.14 ± 0.14, respectively) (Table 2). Differences for Al saturation (%) were found between species (P = 0.0144) where *B*. *pentamera* presented the highest percentage with a mean of 71.80 ± 0.22 and *Z*. *logifolia* presented the lowest percentage with a mean of 21.72 ± 6.46 (Table 2). For exchangeable Al value differences between species (P = 0.0050), *B*. *pentamera* presented the highest content with 8.27 ± 0.78 cmol kg^-1^, and *Z*. *logifolia* presented the lowest content with 0.90 ± 0.78 cmol kg^-1^ (Table 2).

**Table 2:** Differences between tree species for the soil chemical variables (Mean±S.E.).

Tree Species	CEC (cmol kg^-1^)	Exchangeable sodium (cmol kg^-1^)	Available Phosphorus (mg kg^-1^)	pH	Exchangeable aluminum (cmol kg^-1^)		Al saturation (%)
*Andira inermis*	12.15±1.29c	0.12±0.01b	52.17±5.05a	5.42±0.14 a	0.95±0.87c		30.7±7.14cd
*Bellucia pentamera*	25.43±1.29a	0.11±0.01bc	26.74±5.05b	4.62±0.14b	8.27±0.78a		71.93±6.46a
*Guarea guidonia*	16.78±1.29bc	0.09±0.01c	27.88±5.05b	5.01±0.14ab	1.75±0.82bc		46.21±6.75bc
*Psidium guajava*	18.89±1.29b	0.09±0.01c	11.98±5.05b	4.55±0.14b	4.22±0.78bc	58.48±6.46ab	
*Zygia longifolia*	17.4±1.29b	0.16±0.01a	46.52±5.05a	5.14±0.14a	0.9±0.78c	21.72±6.46d	

Equal letters between species indicate equal means (P > 0.05, n = 36). C.E.C: Cation Exchange Capacity.

The cation exchange capacity (CEC) showed differences between species (P = 0.0065), with the highest mean for *B*. *pentamera* of 25.43 ± 1.29 cmol kg^-1^, and *A*. *inermis* with the lowest level with 12.15 ± 1.29 cmol kg^-1^ (Table 2). Exchangeable sodium values presented differences between species (P = 0.0056), where *Z*. *longifolia* showed the highest mean with 0.16 ± 0.01 cmol kg^-1^, and *P*. *guajava* showed the lowest mean with 0.09 ± 0.01 cmol kg^-1^ (Table 2). Available P content presented significant differences (P = 0.0123), where *A*. *inermis* presented the highest mean with 52.17 ± 5.05 cmol kg^-1^, and *P*. *guajava* presented the lowest mean with 11.98 ± 5.05 cmol kg^-1^ (Table 2). Soil organic carbon (SOC), soil organic matter (SOM), calcium (Ca), and exchangeable magnesium (Mg) did not show differences (P <0.05) between species or distance.

### Soil physical characteristics

**Fig 3:**
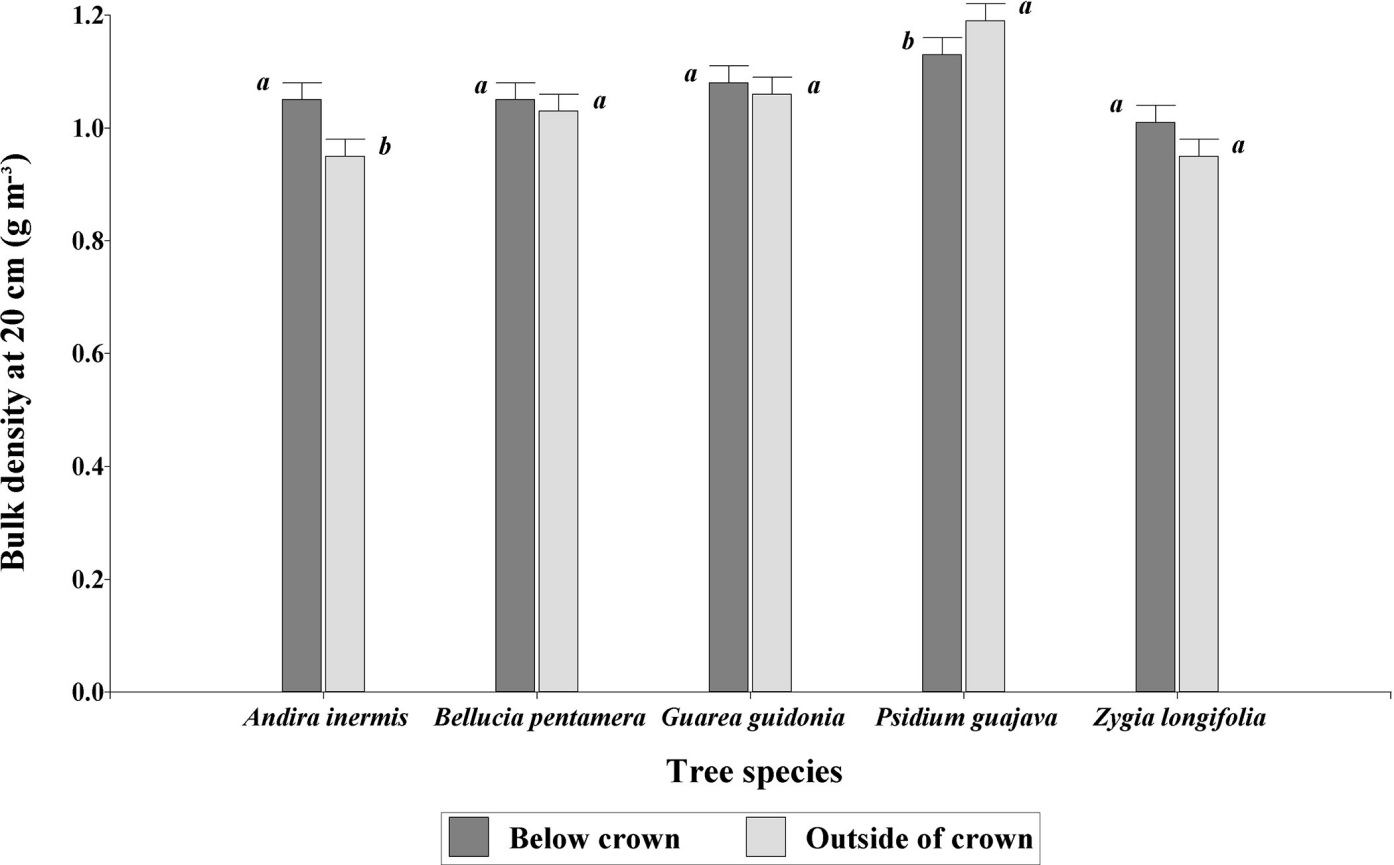
Soil bulk density values in the species-by-distance interaction at 20 cm depth. Equal letters within species indicate equal means (P > 0.05).

The analysis of variance of soil physical variables showed significance only for the interaction distance by tree species for bulk density at 20 cm depth (P = 0.0003). At other soil depths, no interaction or effect of distance or species was found. *Psidium guajava* presented higher soil bulk density values outside of crown (1.19 ± 0.03 g cm^-3^) than below its crown (1.13 ± 0.03 g cm^-3^). *A*. *inermis* presented a higher bulk density under its crown (1.05 ± 0.03 g cm^-3^) than outside of it (0.95 ± 0.03 g cm^-3^). *G*. *guidonia*, *B*. *pentamera* and *Z*. *longifolia* did not show differences in any of the two distances at 20 cm soil depth (Fig 3).

For the variable resistance of the soil to penetration at the three depths evaluated, the interaction between distance by tree species was found (P <0.0001). Greater resistance to the soil penetration was detected under *Z*. *longifolia* (191.47 ± 16.67 kPa cm^-2^) than outside it at 10 cm (96.25 ± 16.67 kPa cm^-2^), at 20 cm (195.83 ± 17.91 kPa cm^-2^ and 106.67 ± 17.91 kPa cm^-2^ respectively), and at 30 cm (197.50 ± 17.66 kPa cm^-2^ and 106.67 ± 17.66 kPa cm^-2^), respectively. The species *G*. *guidonia*, *A*. *inermis*, *P*. *guajava* y *B*. *pentamera* did not show significant differences in the two distances at all depths (Table 3).

**Table 3:** Penetration resistance (Mean±S.E.) in the combination of tree species by distance at three different depth in grazing areas of pastures in the Colombian Amazon.

Tree Species	Soil resistance at 10 cm (kPa cm^-2^)		Soil resistance at 20 cm (kPa cm^-2^)		Soil resistance at 30 cm (kPa cm^-2^)	
	Below crown	Outside of crown	Below crown	Outside of crown	Below crown	Outside of crown
*Andira inermis*	171.88±16.67a	157.02±16.67a	194.38±17.91a	186.5±17.91a	195.27±17.66a	189.63±17.66a
*Bellucia pentamera*	99.88±16.67a	92.23±16.67a	99.88±17.91a	90.12±17.91a	90.25±17.66a	82.27±17.66a
*Guarea guidonia*	181.15±16.67a	171.13±16.67a	200.38±17.91a	193.38±17.91a	213.4±17.66a	215.77±17.66a
*Psidium guajava*	111.83±16.67a	111.42±16.67a	124.58±17.91a	112.58±17.91a	120.17±17.66a	109.5±17.66a
*Zygia longifolia*	191.47±16.67a	96.25±16.67b	195.83±17.91a	106.67±17.91b	197.5±17.66a	106.67±17.66b

Equal letters between species indicate equal means (P > 0.05).

**Fig 4:**
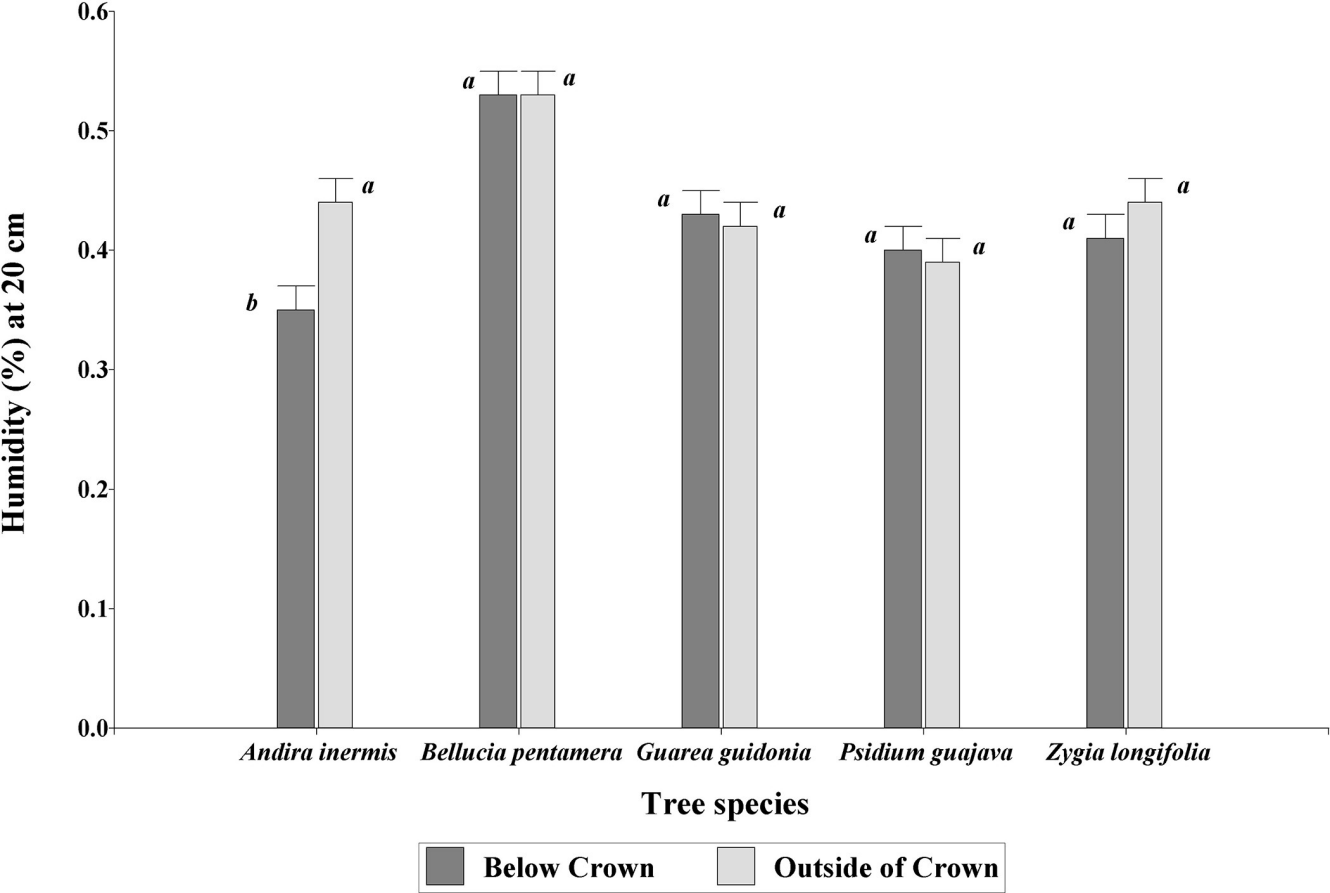
Percentage of soil moisture for the combination of tree species by distance at 20 cm depth in grazing areas of pastures in the Colombian Amazonian piedmont. Equal letters within species indicate equal means (P > 0.05).

The variable moisture at ground level at 20 cm depth showed significant (P = 0.0073) effects between tree species and distance. Moreover, the species evaluated *A*. *inermis* presented a lower percentage of moisture below its crown (0.35 ± 0.02) than outside of it (0.44 ± 0.02), while *P*. *guajaba*, *Z*. *longifolia*, *G*. *Guidonia*, and *B*. *pentámera* did not show significant differences in any distance (Fig 4).

### Relationships between edaphic properties and tree species

**Fig 5:**
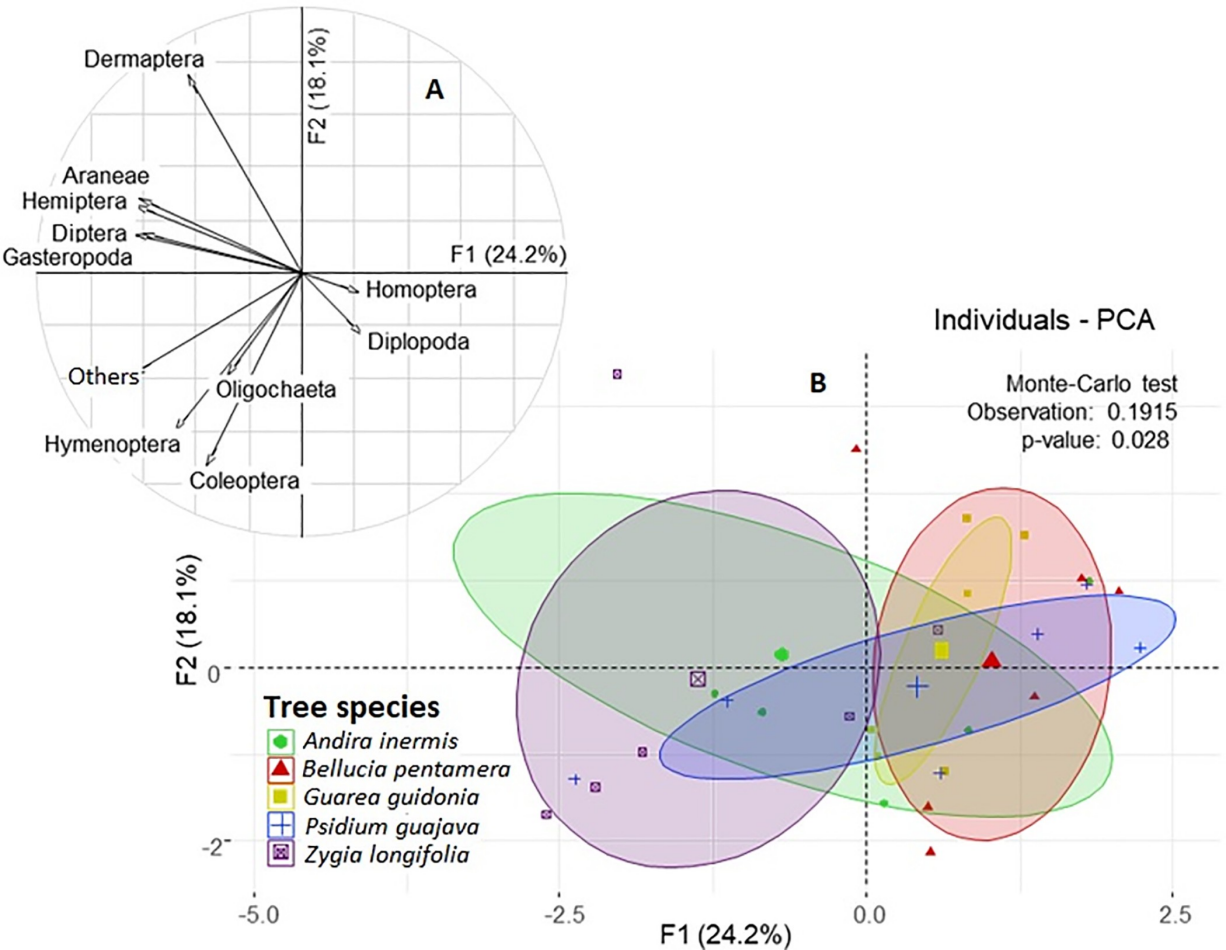
Projection on the factorial plane of the first two axes of a principal component analysis of the edaphic macrofauna variables and of the sampling points grouped according to tree species. A. Correlations of edaphic macrofauna groups. B. Sorting of samples by macrofaunal variables and identified by tree species.

The PCA for the macrofauna groups explained 42.3% of the variability of the data with the first two components. PC_1_ projected the incidence of the crown of each species on the distribution of macrofauna groups. For instance, the soils below the species *Andira inermis* and *Zygia longifolia* were related to Araneae, Hemiptera, Diptera, and Gasteropoda, while those of *Bellucia pertamera* and *Psidium guajava* were related to the Homoptera and Diplopoda. The PC_2_ projected the Dermaptera group in the upper part and the Coleoptera group in its lower part. Both groups did not show a strong relationship with any tree species. The separation of the tree species according to the macrofauna groups was significant, indicating the incidence of the species on the macrofauna (P = 0.0281) (Fig 5).

**Fig 6:**
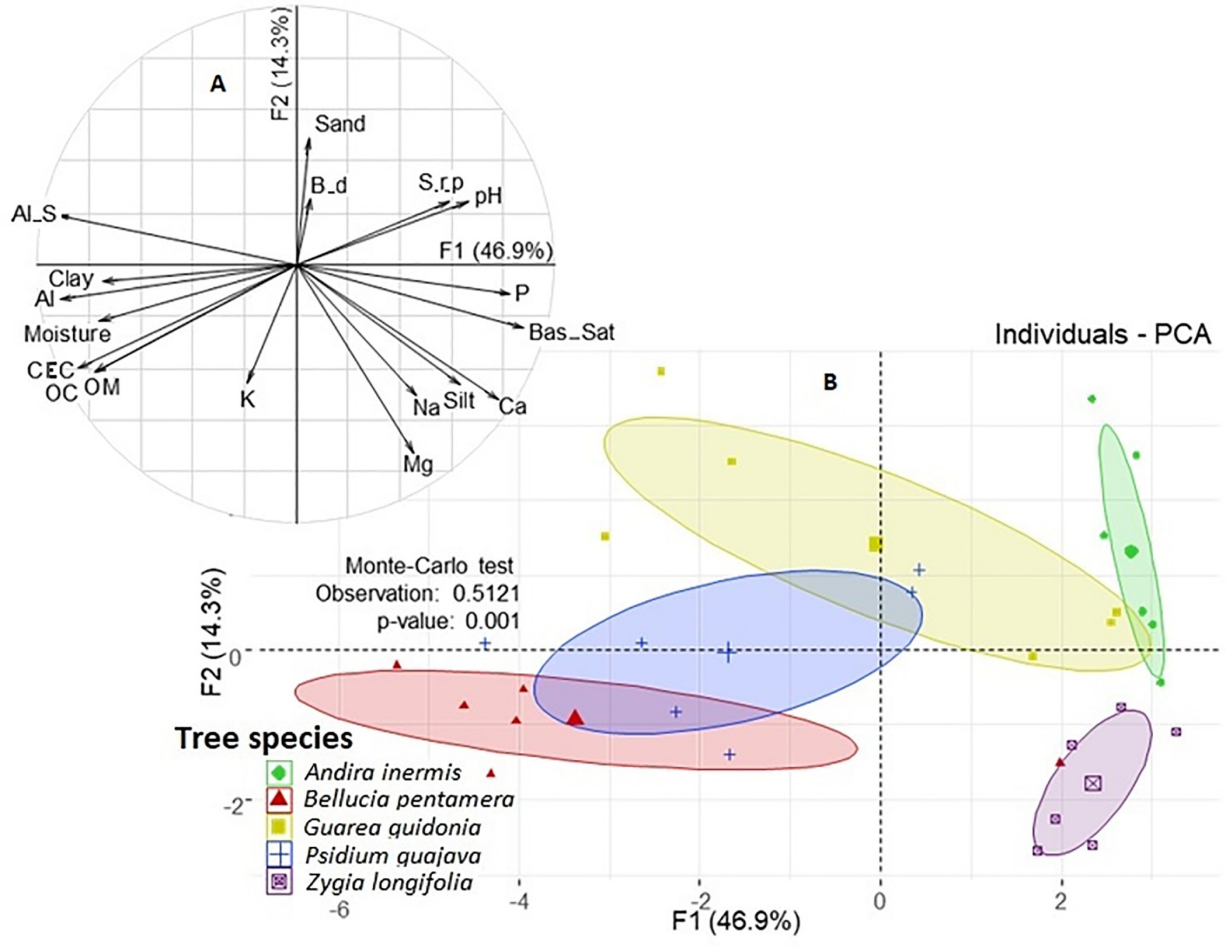
Projection on the factorial plane of the first two axes of a principal component analysis of edaphic variables and sampling points grouped according to tree species. A. Macrofauna correlations. B. Sorting of samples by edaphic variables according to tree species.

The PCA for edaphic properties (physical and chemical) explained 61.2% of the total variability of the data with the first two components. PC_1_ is related to the species *P*. *guajava* and *B*. *pentamera* with the highest Al saturation, moisture, CEC, SOC and SOM; while the species *A*. *inermis* and *Z*. *longifolia* are characterized by having higher values of pH, soil resistance to penetration, available P, exchangeable Ca, and silt. PC_2_ separated the species *Z*. *longifolia* characterized by having the highest levels of exchangeable Mg and Na. The effect under the cover of the species were significant according to the Monte Carlo test (Fig 6).

When investigating the relationships between data sets of tree species using Co-inertia analysis, eight significant correlations were found. All tree species obtained significant relationships when contrasting the chemical and physical properties of the soil, except for *P*. *guajava*. Regardless of the arboreal species, significant relationships were found under the cover when macrofauna data were contrasted with the physical and chemical properties of the soil (Table 4).

**Table 4:** Matrix coefficient (RV) between the three datasets for tree species and location.

Species	Macrofauna—soil chemical		Macrofauna—soil physical		Soil chemical—soil physical	
	RV	p-value	RV	p-value	RV	p-value
*Andira inermis*	0.277	0.433	0.324	0.425	0.618	0.007*
*Bellucia pentámera*	0.453	0.076	0.430	0.166	0.715	0.004*
*Guarea guidonea*	0.387	0.599	0.434	0.448	0.584	0.009*
*Psidium guajava*	0.464	0.112	0.285	0.733	0.391	0.116
*Zygia longifolia*	0.376	0.481	0.428	0.091	0.738	0.001*
Below crown	0.277	0.008*	0.288	0.011*	0.578	0.001*
Outside of crown	0.168	0.418	0.205	0.235	0.606	0.001*

## Discussion

The greater abundance of the macrofauna below the crown of *A*. *inermis*, *G*. *guidonia*, *P*. *guajava*, and *Z*. *longifolia* trees than abundance outside of crown in grazing areas of pastures is presumably due to two factors: the quality of litter and microclimatic conditions. Trees that are present in grazing areas contribute to the SOM of soil due to the entry of senescent leaves, bark, branches, and roots to the system that are decomposed by the macrofauna [48]; and the presence of these individuals depends on the quantity and quality of litter provided by each species [49]. The factor that had the greatest impact on macrofauna populations was the distance, a situation that has also been described for other tree species such as *Croton megalocarpus*, *Eucalyptus grandis* and *Zanthoxylum gilletii* [50]. Likewise, this higher population below the crown is due to traits related to the quality of the leaf litter, which influences the increase of individuals under this position [50].

In our study we found differences in macrofaunal populations according to tree species, a condition that has been a characteristic in different studies. For example, Vohland and Schroth [51] found that the general abundance of fauna was significantly higher in *Bactris gasipaes* and *Bixa orellana* compared to that obtained in *Bertholletia excelsa* and *Theobroma grandiflorum*, because of differences in the quality of plant tissue. Similarly, Gholami et al. [52] found that the abundance and diversity of the macrofauna were spatially related to the density of tree cover, the diversity, and uniformity of the tree species and that they may be related to the microclimate conditions provided by them, showing that the density of trees and their diversity could be the key drivers of the spatial pattern of soil macrofauna diversity.

On the other hand, Laossi et al. [53] have pointed out that the abundance of soil fauna is affected by the quality of the litter and to a greater extent by its quantity. *Zygia longifolia* and *A*. *inermis* presented the highest values in the abundance of the macrofauna under their covers. These two species offer a high level of shade (they have a dense crown), which can probably influence the microclimatic conditions of the soil under their crown. It has been reported in several studies that below the cover of trees under tropical conditions, the temperature is on average 2 to 3°C lower than in open areas [54] and in some sites the difference may reach up to 9.5°C [55], thus affecting air/soil temperature [56, 57] and soil moisture [58, 59]. These conditions are important as many soil organisms are sensitive to soil moisture and temperature regimes [60].

The importance of trees in grazing areas was significant as below canopy populations of macrofauna increased significantly compared to those in other production systems. These data are consistent with those obtained by Durán et al. [61] and Rodríguez et al. [12] who carried out their sampling without considering the effect of the influence of the tree canopy on pastures that are in a certain state of degradation. Therefore, increasing the levels of shade in pastures improves different ecosystem services [26, 62, 63] related mainly to the contribution of biomass [64] (quantity and chemical composition), microclimatic conditions [62, 65] (humidity and temperature) that affect the richness and density of the soil macrofauna community [66]. In this sense, increasing the density of trees in pastures specifically in silvopastoral systems increases macrofauna populations [64, 67].

Rhoades [48] states that scattered trees in pastures affect the chemical conditions of the soil, due to the entry of organic matter from leaves, bark, branches, and roots to the soil system, which the soil fauna transforms and decomposes. This effect occurs in the same way for *P*. *guajava* with Mg^+^ and K^+^, and for *Z*. *longifolia* with K^+^, where the concentration of these elements increases under the area of influence of their crowns. These results agree with those made by Dahlgren et al. [68] in blue oak (*Quercus douglasii*), by Eldridge and Wong [69] in four species of *Eucalyptus* sp., in a temperate zone of Australia, by De Boever et al. [70] in an arid ecosystem in Tunisia, with *Acacia raddiana* trees, and those made by Kooch et al. [71] in the north of Iran. In general, it demonstrates the importance of scattered trees in pastures to increase soil fertility, being this, among other ecosystem services offered by trees in grazing areas [72].

Casals et al. [28] evaluated differences between the effects of legume and non-legume tree species on soil nutrients and carbon reserves in pastures in Nicaragua, finding higher levels of SOC, N, P, K^+^, and Ca^2+^ under the tree cover than in open field pastures, regardless of whether they were legumes or non legumes, concluding that the magnitude of the effect depends more on tree characteristics such as basal area and crown area than on whether or not the species is a legume. Thus, when relating the effect of trees, De Boever et al. [70] found higher concentrations of nutrients in the soil under the crown of the tree: 175% more compared to outside the crown. However, other biotic and abiotic factors have been associated with the effects of individual plants on soil properties, such as plant species [73], age [70], topography [74], soil texture [75] and functional traits of the tree species [29, 76] and the rate of fall and decomposition of litter [77, 78]. In other types of ecosystems Avendaño-Yáñez et al. [79], Kumar et al. [80], and Mohammed et al. [81] observed that trees improve the fertility of soils under their crowns. We found a significant increase in exchangeable potassium under crown *Psidium guajava* and *Zygia longifolia*.

The high values of soil bulk density observed under the crown of some species (*A*. *inermis* and *Z*. *longifolia*) are mainly due to the use given by the animals as resting areas, where the soil receive a pressure of 1.2 to 1.6 kg cm^-2^ [82]. This increase in soil bulk density was related to high values in soil resistance to penetration under its crown. Comparing our results with those presented by Frost and Edinger [83] and Dahlgren et al. [68] they report that they were lower bulk density values. These differences are probably due to the continuous stocking as well as the tree species found in the paddocks referenced in the previous studies. For example, Frost and Edinger [83] report lower bulk density values under *Quercus wislizenii* crowns than in open grasslands, as well as Dahlgren et al. [68] who investigated the *Quercus douglasii* species under different management combinations: *Q*. *douglasii* with stocking, *Q*. *douglasii* without stocking, open grasslands with stocking and open grasslands without stocking; where the soils that are under the crown of *Q*. *douglasii* presented a lower value of bulk density. Likewise, Tate et al. [84] found that crown cover by any tree species they evaluated in their research significantly reduced the bulk density of the soil surface (16 to 22%) compared to open grasslands. Kumar et al. [80] in an arid region found that the bulk density values under three species (*Prosopis cineraria*, *Acacia Senegal*, and *Tecomella undulata*) were lower than in the open field. Differences observed in bulk density from the studies here cited might be the result of different climatic conditions driving the behaviour of the animal occupying the paddocks thus causing different compaction patterns. *A*. *inermis* and *Z*. *longifolia* are tree species that have dense and large crowns that provide areas of greater shade at ground level, allowing grazing cattle to seek these thermoregulation zones to lower body temperature due to high temperature that occurs in the study area (25.5°C). Moreover, the animals seek the grasses that grow under these areas for their nutritional quality and sometimes the forage (leaves, flowers, and fruits) provided by the trees. Similarly, another factor that can affect this physical variable is the precipitation in the study area (3,793 mm).

Wilson [85] found that along 20 m long transects extending from the crown of trees of the *Eucalyptus melliodora*, *Eucalyptus blakelyi*, and *Eucalyptus nova-anglica*, species, the bulk density increased significantly as distance increased relative to the canopies of the trees. In contrast, Kooch et al. [71] in an agroecosystem different from this research work, evaluated the bulk density affected by trees in a mixed forest in Northern Iran, finding an increase in the bulk density of the soil under trees of the *Carpinus betulus* species, due to the low presence of SOM in the soil.

The species *A*. *inermis* and *Z*. *longifolia* are trees that have morphological and crown characteristics that allow greater shade at ground level. This allows pastures to have a high concentration of animals in shady sites, negatively affecting the physical properties of the soil and especially the bulk density with high levels of compaction. This can lead to a reduction in root density [86] as well as the volume of soil pores and consequently, the infiltration of water [87, 88] at different depths of the soil. This explains the results found in this work, where the percentage values of soil moisture were higher outside the tree crown than under it.

Stocking density in our study was two animals ha^-1^. Schmalz et al. [89] found an increment in the penetration resistance when comparing paddocks with different stocking densities, penetration resistance was higher in paddocks with 1.56 animals ha^-1^ compared to those with 0.52 animals ha^-1^. Additionally, Greenwood & McKenzie [88] concluded that a stocking density of 1.9 to 2.4 animals ha^-1^ increases compaction and bulk density and decreases soil infiltration in the first 20 cm. Consequently, farm management decisions must take into consideration stocking density [89], particularly in climatic regions were animals due to high temperatures tend to seek refuge under the tree crowns increasing compaction.

We provide evidence that the diversity and density of macrofaunal populations change under the canopy as well as at the tree species level. Thus, increasing shade canopies in paddocks increases the sustainability of livestock production systems. Trees in paddocks provide favorable conditions for macrofauna by increasing the amount of leaf litter in the soil, as well as the chemical composition of the litter [90]. Therefore, as evidenced by the results of the co-inertia analysis, by increasing macrofauna populations there is a significant effect on soil chemical (available P, K, Na, CEC, pH, Al saturation) and soil physical characteristics (bulk density, soil moisture and penetration resistance). Results from this study also demonstrated how trees in the grazing areas of the pastures in the Colombian Amazon increased some regulation of ecosystem through improved soil fertility [91].

## Supporting information

S1 File Physical, chemical, and biological variables of soil in grazing areas. (XLSX)
